# Combining Stimulation Protocols to Probe Memory Consolidation—Commentary on Hausdorf et al. 2025

**DOI:** 10.1111/ejn.70400

**Published:** 2026-01-19

**Authors:** Laurent Sheybani

**Affiliations:** ^1^ Neurology, Department of Clinical Neuroscience University Hospitals of Geneva Geneva Switzerland

**Keywords:** brain stimulation, electrophysiology, memory

## Abstract

A recent study by Hausdorf et al. reported that combining closed‐loop acoustic stimulation with transcranial direct current stimulation during sleep does not improve memory consolidation. This contrasts with past studies that reported a beneficial effect of each method individually. In this commentary, I discuss the relevance of the study in the current literature and offer potential explanations for this absence of effect.

## 1 Main

In 2007, a team led by Jan Born at the University of Lübeck in Germany published a pioneering study demonstrating the possibility to manipulate memory consolidation during sleep (Rasch et al. 2007). By associating an odor to the learning phase of an object‐location memory task, they showed that re‐exposure to the same odor during subsequent slow‐wave sleep (SWS) significantly enhanced memory performance the following day. This study opened the field of neurostimulation to sleep science, and numerous studies using brain stimulation paradigms followed over the subsequent years (Bellesi et al. 2014; Lustenberger et al. 2016; Fehér et al. 2021).

SWS—the sleep stage targeted by Rasch and colleagues in 2007—is defined by the presence of at least 20% slow‐wave activity (SWA) (Troester et al. 2023). SWA refers to rhythmic oscillations within the 0.5–2 Hz frequency range, with amplitudes exceeding 75 μV as recorded via scalp electrodes (Troester et al. 2023). These oscillations reflect the repetition of slow waves (SW), which are fluctuations in neuronal membrane potential between depolarized (UP) and hyperpolarized (DOWN) states (Timofeev et al. 2020). Although SWs predominantly occur during sleep, they have also been observed during wakefulness in healthy individuals (Andrillon et al. 2021) and people with epilepsy (Sheybani et al. 2023; Sheybani, Frauscher, et al. 2025; Sheybani, Vivekananda, et al. 2025). Among their various functions, SWs play a critical role in memory consolidation, particularly through their coordination with sleep spindles and sharp‐wave ripples—two key phenomena involved in memory processing (Huber et al. 2004; Rasch and Born 2013; Bush et al. 2022; Staresina et al. 2023).

Building on the understanding that SWs contribute to memory consolidation, several research groups have attempted to enhance this process by modulating SWA during sleep (Fehér et al. 2021). Techniques such as acoustic, electrical, and magnetic stimulation have been employed to augment SWA and assess its effects on memory. However, results have been mixed, and the underlying reasons remain incompletely understood. One possible explanation is that the efficacy of stimulation may depend on the brain's ongoing activity at the time of intervention.

Evidence increasingly supports the notion that brain function is context‐dependent (Russo et al. 2025). In vitro studies have shown that background network activity strongly influences synaptic plasticity mechanisms (Reig et al. 2006). This aligns with the well‐established observation that identical stimuli can elicit different neural responses depending on the brain's current state (Arieli et al. 1996). Such variability may be partially attributed to fluctuations in cortical excitability across the sleep–wake cycle (Ly et al. 2016). Consequently, the impact of SWA enhancement may vary depending on the brain's activity during sleep.

A recent study by Hausdorf et al. 2025, published in the *European Journal of Neuroscience*, tested this hypothesis in a cohort of 20 participants. Subjects completed three memory tasks before spending two nights in a sleep laboratory. The authors used closed‐loop acoustic stimulation to enhance SWA. Then, to test the impact of ongoing brain activity in shaping response to auditory stimuli, participants also received transcranial direct current stimulation (tDCS) on one of the nights (pseudo‐randomized and counterbalanced). tDCS occurred as two 15‐min sessions restricted to non‐rapid eye movement (NREM) sleep Stage 3. The authors hypothesized a synergistic effect, expecting that the known benefits of acoustic stimulation would be amplified by the increased cortical excitability induced by tDCS.

Contrary to expectations, memory performance did not differ between the acoustic stimulation condition alone and the combined acoustic + tDCS condition. Interestingly, when participants were stratified by cognitive ability, those with higher cognitive performance exhibited reduced memory consolidation with tDCS, whereas those with lower cognitive performance showed no significant change. While baseline memory performance after sleep (i.e., sham condition without acoustic stimulation or tDCS either) would have been interesting for interpretation of these results, this unexpected outcome raises important questions.

In the study, SWs were detected using a closed‐loop algorithm applied to a frontal EEG derivative (Fpz). However, SWs are known to be focal or multifocal phenomena (Nir et al. 2011). Thus, the effectiveness of closed‐loop stimulation may depend on the simultaneous generation of SWs in both frontal and temporomesial regions—an interaction believed to be crucial for optimal memory consolidation (Rasch and Born 2013). Supporting this, recent work by Yuval Nir and Itzhak Fried (Geva‐Sagiv et al. 2023) suggests that closed‐loop stimulation targeting frontally‐generated SWs can enhance memory, but only if the stimulation is also aligned with hippocampal SWs. This may explain the lack of overall benefit observed in the study, though it does not account for the differential effects based on cognitive ability. Related to this, another potential confounder is that closed‐loop stimulation was not triggered by SW phase, but by the mean latency between SW negative to positive peak. This procedure can introduce variability in the EEG response, in comparison to a stimulation triggered by SW phase (Esfahani et al. 2023), thus potentially contributing to a lack of effect.

In earlier work by the same group, tDCS alone was shown to improve memory consolidation by increasing SWA amplitude (Marshall et al. 2004). Acoustic stimulation typically induces a train of SWs following a detected wave (Ngo et al. 2013). When combined with tDCS, this may thus disrupt the delicate coordination required for SWA to propagate effectively across the fronto‐temporal network. Indeed, instead of supporting communication between the temporal and frontal areas, this may actually trigger SWs at detrimental times. Precise coordination of sleep entities (SW, spindles, and sharp‐wave ripples) is crucial for normal brain processing (Helfrich et al. 2018; Muehlroth et al. 2019). Morphological changes (e.g., increased length of SWs) induced by the intervention could also have contributed to altered coordination of key entities involved in memory consolidation.

Last but not least, while both tDCS (Marshall et al. 2004) and closed‐loop acoustic stimulation (Ngo et al. 2013) have independently been shown to enhance memory consolidation, other studies have also reported a lack of or minimal behavioral effect (Henin et al. 2019; Wunderlin et al. 2021) despite various neurophysiological impacts (Wunderlin et al. 2021). Furthermore, even if one concedes that these procedures succeed consistently, it cannot be assumed that their combination will yield additive benefits. Indeed, the brain is not governed by linear processes; a typical example is the substantial increase in superior colliculi cells' response to multisensory inputs, largely exceeding the sum of the responses to each individual sensory modality (Meredith and Stein 1986). Nevertheless, the innovative approach of Hausdorf et al. (2025) is particularly valuable in the context of a negative result, as it enables investigation into the mechanisms by which these two proven (or, at least, supposedly) beneficial interventions may interact antagonistically.

Thus, elucidating how temporally and frontally generated SW and spindles are modulated by each intervention alone, and then in combination, is essential to clarify the mechanisms underlying the adverse effects of tDCS combined with acoustic stimulation in high‐performing individuals.

One possibility would be to investigate the effects of acoustic stimulation, with and without tDCS, using intracranial recordings. The hypothesis is that acoustic stimulation superimposed on tDCS may lead to asynchronous SWA between these regions. Individuals with lower cognitive performance may not exhibit a decline due to a floor effect.

Intracranial recordings could also provide more detailed insights into SW morphology and associated neural activity. In rodent models, it has been shown that whether an UP‐state is preceded by a DOWN‐state or not can determine whether memory is enhanced or disrupted (Kim et al. 2019). Moreover, although NREM 3 is characterized by intense SWA, other, less understood neural activities also contribute to this state. Notably, even when SWA is suppressed in EEG recordings, NREM 3 can still be identified based on neuronal activity alone (Parks et al. 2024). Therefore, combined acoustic and electrical stimulation may have unforeseen effects on these additional NREM 3‐specific processes that also support memory consolidation.

In conclusion, the study by Hausdorf et al. (2025) presents a conceptually innovative and technically challenging approach to probing the role of SWA in memory consolidation. While the lack of a synergistic effect between acoustic stimulation and tDCS was unexpected and the detrimental impact on high cognitive performers particularly intriguing, the findings open new avenues for research. This work introduces a novel paradigm for perturbing and investigating SWA‐related mechanisms during sleep, with important implications for understanding and enhancing memory consolidation.

## Author Contributions

**Laurent Sheybani:** funding acquisition, conceptualization, resources, writing ‐ original draft, writing ‐ review and editing.

## Funding

This work was supported by the Swiss National Science Foundation (SNSF), Grant No. PZ00‐3_233452, and the Guarantors of Brain.

## Conflicts of Interest

The author declares no conflicts of interest.

## Data Availability

This work did not generate any data.
